# Exhaled Breath Condensate in Childhood Asthma: A Review and Current Perspective

**DOI:** 10.3389/fped.2019.00150

**Published:** 2019-04-25

**Authors:** Michiel A. G. E. Bannier, Philippe P. R. Rosias, Quirijn Jöbsis, Edward Dompeling

**Affiliations:** ^1^Department of Pediatric Respiratory Medicine, School for Public Health and Primary Care (CAPHRI), Maastricht University Medical Center, Maastricht, Netherlands; ^2^Department of Pediatrics, Zuyderland Medical Center, Sittard, Netherlands

**Keywords:** asthma, wheeze, exhaled breath condensate, exhaled breath, children

## Abstract

Exhaled breath condensate (EBC) was introduced more than two decades ago as a novel, non-invasive tool to assess airway inflammation. This review summarizes the latest literature on the various markers in EBC to predict asthma in children. Despite many recommendations and two comprehensive Task Force reports, there is still large heterogeneity in published data. The biggest issue remains a lack of standardization regarding EBC collection, preservation, processing, and analysis. As a result, published studies show mixed or conflicting results, questioning the reproducibility of findings. A joint, multicenter research study is urgently needed to address the necessary methodological standardization.

## Introduction

More than two decades ago, exhaled breath condensate (EBC) was introduced as a novel non-invasive diagnostic technique for various respiratory diseases. EBC is obtained by cooling exhaled breath, and mainly contains water vapor and a small fraction of volatile and non-volatile macromolecules (1, 2). These include various markers of airway inflammation, such as acidity (pH), hydrogen peroxide (H_2_O_2_), nitrogen oxides (NO_x_), eicosanoids, and cytokines (1, 2). In children, non-invasive assessment of airway inflammation is of pivotal importance as current gold standards (bronchoalveolar lavage and endobronchial biopsy) are invasive and therefore not suitable for routine use. Although airway inflammation is a hallmark of asthma, this phenomenon is currently not used as a diagnostic feature (3). Despite well-defined diagnostic criteria and the availability of lung function tests, both underdiagnosis and overdiagnosis of asthma still frequently occur (4). This is particularly relevant in preschool children with wheezing symptoms (5). Therefore, the availability of reliable non-invasive biomarkers of airway inflammation has been suggested to overcome this diagnostic issue. Moreover, other potential applications include the monitoring of asthma, the prediction of asthma exacerbations, and guidance in treatment decisions. At the end of the last millennium, EBC was introduced as a non-invasive tool with high potential for the diagnosis and monitoring of various inflammatory airway diseases, including asthma. Despite two comprehensive Task Force reports of the European Respiratory Society (ERS) and American Thoracic Society (ATS) in 2005 and 2017, respectively (1, 2), there has not yet been a significant clinical breakthrough, nor the onset of methodological standardization.

In this review, we aim to provide a concise update on markers in EBC to predict asthma in children. For this purpose, the PubMed database was searched for relevant publications, with at least an English abstract, in the last 5 years (January 2013–December 2018). For review of EBC literature older than 5 years, we refer to two other excellent reviews (6, 7). All different applied analytical methods of EBC and their limitations were reviewed by Rahimpour et al. (8). True methodological studies remain scarce and fall beyond the scope of this article, as do other review articles.

## Methodological Heterogeneity

A total of 30 studies with original data in the past 5 years were identified, of which 20 had a cross-sectional design (Table 1) and 10 a longitudinal approach (Table 2). As has been the case with older EBC studies, these studies showed great heterogeneity regarding study population, type of biomarkers, and methodology used to collect, preserve, and analyze EBC. Study groups ranged from children with mild to severe asthma, preschool wheezers to adolescents, and controlled to uncontrolled asthma. Furthermore, the diagnosis of asthma differed between studies and was not always in accordance with international guidelines. Moreover, various asthma phenotypes were studied including non-atopic and atopic asthma, obesity-related asthma, and exercise-induced asthma. For the collection of EBC, 5 different condenser systems were used of which the EcoScreen was most frequently applied (15/30 studies). The most frequently studied EBC biomarkers were cytokines (*n* = 10), acidity (*n* = 9), and eicosanoids (including 8-isoprostane, cysteinyl-leukotrienes, and lipoxins: *n* = 9). For measurement of the various biomarkers, 14 different analytical methods were used (counting the different ELISA techniques as one, the different enzyme immuno-assays as one, and the different multiplex immuno-assays as one).

**Table 1:** Cross sectional studies using EBC in children with asthma.

**References**	**Study population**			**EBC biomarker**		**EBC methodology**		
	**Age (years)**	**Study groups**	***N***	**Marker**	**Outcome (*p*-value)**	**Condenser**	**Rate of detection**	**Analysis**
Aldakheel et al. (9)	17.8 (mean)	Atopy cohort study •poly-aero-sensitization •mono-aero-sensitization •atopic asthma •atopic rhinitis	361 •179 •54 •72 •111	NOx	•increased (0.01) •NS •increased (0.04) •NS	Glass (Custom-made)	48%	Fluorescent modification of Griess
Benor et al. (10)	8–16	•Asthma •Healthy	•37 •15	UFP (PM_0.1_)	•NS between groups •Correlation with wheezing (0.04), breath symptom score (0.03), sputum eosinophilia (0.005)	Turbo-Deccs	n/a	Nanoparticle Tracking Analysis (NanoSight light microscope LM20)
Bodini et al. (11)	6–14	•Obese, non-asthma •Obese, asthma •Non-obese, asthma •Healthy	•20 •11 •15 •15	Leptin	•increased in obese children (<0.001) •increased in asthma vs. healthy (0.05)	Turbo-Deccs	NR	ELISA (BioVendor)
Brzozowska et al. (12)	8–17	Severe asthma	8	Cytokines	•positive correlation between FeNO and IL-2, MCP-1, PDGFBB, TIMP-2 in EBC	EcoScreen 2	100% for 13 cytokines >50% for 20 cytokines	Multiplex ELISA array (Ray Biotech)
Carraro et al. (13)	8–17	•Nonsevere asthma •Severe asthma •Healthy	•31 •11 •15	Metabolomic profiling	•Discrimination severe asthma vs. healthy: AUC of O2PLS-DA model: 1.00 •Discrimination severe asthma vs. nonsevere asthma: AUC of O2PLS-DA model: 0.83 •Compounds relevant for discrimination were related to retinoic acid, adenosine and vitamin D	Turbo-Deccs	n/a	Ultimate 3000 HPLC system (Dionex) coupled to LTQ Orbitrap mass spectometer (Thermo Fisher)
Grzela et al. (14)	13.2 (mean)	•Severe asthma •Mild asthma •Healthy	•6 •8 •9	TSP-1, DPP-IV, MMP-9, TIMP-1, and ANG	•increased in severe asthma vs. healthy and mild asthma (calculated as relative expression index)	EcoScreen	100%	Human Angiogenesis Array (R&D Systems)
Grzela et al. (15)	13.4 (mean)	Mild allergic asthma •MMP-9 polymorphisms: genotypes Q279R and R668Q	20	MMP-9	•highest levels in subjects with both polymorphisms •higher concentrations using ELISA vs. activity assay	EcoScreen	NR	•QuickZyme Human MMP-9 Activity Assay (BioSciences) •Human MMP-9 Quantikine ELISA Kit (R&DSystems)
Keskin et al. (16)	6–18	Asthma:•mild persistent •moderate persistent	30: •19 •11	•Cys-LTs •8-IP	•Cys-LTs NS •8-IP increased in moderate persistent asthma (0.05) and in >4 exacerbations/year(<0.05)	EcoScreen	•73% •100%	EIA (Cayman)
Linares Segovia et al. (17)	7–12	City children, exposure to high levels of air pollution (PM_10_) •asthma •non-asthma	103: •51 •52	Cys-LTs	•increased in asthma vs. healthy (0.0005) •increased in persistent asthma vs. intermittent asthma (0.02) •increased in persistent asthma vs. healthy (0.001)	RTube	100%	EIA (Cayman)
Linares Segovia et al. (18)	7–12	•Asthma •Healthy	•51 •52	IL-6	•increased (0.0001)	RTube	100%	EIA (BioSource)
Majak et al. (19)	8–19	Asthma and EIB symptoms: •positive ETC •negative ETC	25: •10 •15	cytokines	•increased MCP-1- and IL-16 (0.022 and 0.017, adjusted to pre-EBC FEV_1_)	EcoScreen 2	NR	Multiplex ELISA array (Ray Biotech)
Maloča Vuljanko et al. (20)	5–7	Asthma-like symptoms: •asthma •asthma with GERD •non-asthma	75: •10 •49 •16	•Single: pH, pCO_2_, pO_2_, Mg, Ca, Fe, urates •Predictive model of 6 markers (Ca excluded)	•NS for each single marker •for asthma diagnosis: AUC 0.698, PPV 84%, NPV 39%, sens 81%, spec 44% (0.046)	EcoScreen	NR	Deaerated pH (argon), Colorimetry, Spectrophotometry
Navratil et al. (21)	6–18	Asthma: •uncontrolled •controlled	103: •53 •50	•pH •urates	•decreased in NCA (0.002) •decreased in NCA (<0.001) •urates best single predictor of asthma control (<0.001): •AUC 0.927, sens 94%, spec 81%, PPV 83%, NPV 94%	EcoScreen	NR	Deaerated pH (argon), Colorimetry, spectrophotometry
Sinha et al. (22)	9.5–10.3 (mean)	•Asthma •Healthy adult controls	•89 •20	NMR spectrum pattern	•differentiation between asthma and healthy: sens 80%, spec 75%	RTube	n/a	NMR spectroscopy (Bruker)
Stelmach et al. (23)	12–18	Asthma and post-exercise symptoms:•MCT^−^ECT^−^ •MCT^−^ECT^+^ •MCT^+^ECT^−^ •MCT^+^ECT^+^	40 •10 •10 •10 •10	•cytokines •eicosanoids	•detectable IL-4 in MCT+ •detectable eicosanoids in ECT+	Ecoscreen	50%	multiplex immunoassay (Millipore) •ELISA (Cayman)
Tahan et al. (24)	5–17	•Asthma with EIB •Asthma without EIB	•17 •28	LipoxinA4	•increased post-exercise in EIB positive asthma (0.05) •no differences in EIB negative asthma •no difference between atopic and nonatopic asthma	RTube	100%	ELISA (Eagle BioScience Inc)
Tahan et al. (25)	5–17	•Asthma with EIB •Asthma without EIB	•11 •7	AnnexinA5	•higher pre-exercise in EIB negative asthma (<0.05) •no difference post-exercise •no difference pre- and post-exercise within groups	RTube	100%	ELISA (EBioScience)
Trischler et al. (26)	9–17	•Asthma •Healthy	•33 •35	LTB_4_	•increased in small airway or alveolar fraction (0.003) •NS in large •Increased (small airway/alveolar fraction) in children with FEV_1_ <-1.65 *z*-score (0.04) *z*-score (0.04) •increased in asthma without obstructive lung function (0.01)	ECoScreen 2	NR	EIA (Cayman)
Turkeli et al. (27)	7–12	•Atopic asthma •non-atopic asthma	•37 •37	•IL-5 •IL-8 •MMP-9	•increased in atopic asthma (0.04) •NS •NS	EcoScreen	NR	ELISA (R&D Systems)
Yan et al. (28)	6–13	•Atopic asthma •Atopic non-asthma	•22 •16	pH	No differentiation between study groups and no association with *Der p 1* exposure	EcoScreen	n/a	Deaerated pH (argon)

*8-IP, 8-isoprostane; ANG, angiogenin; AUC, area under the receiver operating characteristics curve; Ca, calcium; Cys-LTs, cysteinyl-leukotrienes; Der p 1, Dermatophagoides pteronyssinus 1 allergen; DPP-IV, dipeptidyl peptidase-IV; EBC, exhaled breath condensate; ECT, exercise challenge test; EIA, enzyme immuno-assay; EIB, exercise-induced bronchoconstriction; ETC, exercise treadmill challenge; Fe, iron; FeNO, fractional exhaled nitric oxide; FEV_1_, forced expiratory volume in 1 second; GERD, gastro-esophageal reflux disease; IA, immuno-assay; IL, interleukin; LTB_4_, leukotriene B_4_; MCP, monocyte chemoattractant protein; MCT, methacholine challenge test; Mg, magnesium; MMP-9, matrix metalloproteinase-9; n/a, not applicable; NCA, uncontrolled asthma; NMR, nuclear magnetic resonance; NOx, nitric oxide products; NPV, negative predictive value; NR, not reported; NS, no significant difference; O2PLS-DA, bidirectional-orthogenal projections to latent structures-discriminant analysis; pCO_2_, carbon dioxide tension; PDGFBB, platelet-derived growth factor BB; pO_2_, oxygen tension; PM_10_, particulate matter ≤ 10 micrometers in diameter; PPV, positive predictive value; sens, sensitivity; spec, specificity; TIMP, tissue inhibitor of metalloproteinases; TSP-1, thrombospondin-1; UFP, ultrafine particle*.

**Table 2:** Longitudinal studies using EBC in children with asthma.

**References**	**Study population**			**EBC biomarker**		**EBC methodology**		
	**Age** **(years)**	**Study groups and Study design**	***N***	**Marker**	**Outcome (*p* value)**	**Condenser**	**Rate of detection**	**Analysis**
Caffarelli et al. (29)	•9.3 •5.1 (mean)	•Asthma exacerbation •Healthy EBC before and 7 days after standard asthma exacerbation treatment	•12 •27	pH	•decreased in asthma vs. healthy before treatment (0.03) •NS after treatment	Turbo-Deccs	n/a	Deaerated pH (argon)
Grzela et al. (30)	•14.0 •12.9 (mean)	•Asthma exacerbation •Healthy Proof of concept, impact of increased ICS doses for 4 weeks, EBC before and after 4 weeks	•4 •6	•MMP-9 •TNF •IL-8	•increased in asthma vs. healthy, no change after increasing ICS •increased in asthma vs. healthy, noticeable decrease after increasing ICS •N/A	EcoScreen	•100% •100% •0%	•QuickZyme Human MMP-9 Activity Assay (BioSciences) •ELISA (Invitrogen) •ELISA (Invitrogen)
Keskin et al. (31)	6–18	Moderate and severe asthma exacerbation:•high-dose ICS •OP Prospective cohort EBC before, 4 h and 6 days after start treatment	52: •34 •18	•Cys-LTs •8-IP	•decreased after 4 h and after 6 days in ICS group (0.001 and <0.0001); decreased after 4 h and after 6 days in OP group (0.012 and 0.018) •decreased 6 days after R/ in severe asthma group (0.023)	EcoScreen	•88% in ICS and 44% in OP group •100%	EIA (Cayman)
Keskin et al. (32)	0.3–3.0	Preschool wheezing:•montelukast •placebo Randomized DBPCS for 56 days Only baseline EBC	62: •30 •32	Eicosanoids:•Lipoxin A4 •PGE2 •Cys-LTs	Only baseline EBC:•NS •NS •increased in montelukast group (0.002)	EcoScreen	NR	ELISA (Cayman)
Klaassen et al. (33)	2–4	preschool wheezing:•asthma at age 6 years •transient wheeze Prospective cohort Follow-up until age 6 years	198: •76 •122	•pH •Cytokines	•No added value in prediction of asthma when added to API (AUC 65%)	Glass (custom-made)	98%	•Non-deaerated pH •multiplex immunoassay (Luminex)
Lozano et al. (34)	6–14	Effect of SIT in allergic asthma:•SIT s.c. (house dust mite) •controls: symptomatic treatment Randomized Prospective cohort EBC before, 4 and 8 months after start	41: •21 •20	pH Variable ratio = pH after/before treatment	•SIT: decreased at 4 and 8 months (0.011 and 0.019) •controls: decreased at 4 and 8 months (0.037 and 0.001) •SIT: increased after 8 vs. 4 months (0.04)	EcoScreen	n/a	Deaerated pH (helium)
Stelmach et al. (35)	12–18	Asthma and post-exercise symptoms, effect of treatment on EIB: •ICS •double dose ICS •ICS+LTRA •ICS+LABA Randomized DBPCS 8 weeks, EBC before and after ECT before, and 8 weeks after intervention	80 •20 •20 •20 •20	•cytokines •PGE2, LTB_4_ and Cys-LTs	Due to low concentrations outcome NR	EcoScreen	NR (detectable in a few subjects)	•multiplex immunoassay (Millipore) •ELISA (Cayman)
Van Vliet et al. (36)	6–18	Asthma, prediction of exacerbations Prospective observational cohort 1 year, EBC every 2 months	96	•pH •Cytokines	•Low predictive power of EBC markers alone (AUC 47%), or when combined with FeNO and clinical characteristics (AUC 59%)	Glass (custom-made)	•n/a •large variability	•Non-deaerated pH •high-sensitivity bead-based flow immunoassay (Millipore)
Van Vliet et al. (37)	6-18	Asthma:•Association breath markers and asthma control •Discrimination persistently controlled vs. uncontrolled asthma Prospective observational cohort 1 year, EBC every 2 months	96	•pH •cytokines	•No association between EBC markers and asthma control (AUC 49%)	Glass (custom-made)	n/a	•Non-deaerated pH •high-sensitivity bead-based flow immunoassay (Millipore)
Wood et al. (38)	3–10	Persistent asthma: •*Mycoplasma pneumoniae +* •*Mycoplasma pneumoniae −* Prospective cohort Up to 8 visits in 24months	31: •20 •11	pH	NS	RTube	n/a	Deaerated pH (argon)

*API, asthma predictive index; AUC, area under the receiver operating characteristics curve; Cys-LTs, cysteinyl-leukotrienes; DBPCS, double-blinded placebo-controlled study; EBC, exhaled breath condensate; ECT, exercise challenge test; EIA, enzyme immuno-assay; EIB, exercise-induced bronchoconstriction; IL, interleukin; ICS, inhaled corticosteroids; LABA, long-acting beta-agonist; LTB_4_, leukotriene B_4_; LTRA, leukotriene receptor antagonist; MMP-9, matrix metalloproteinase-9; n/a, not applicable; NR, not reported; NS, no significant difference; OP, oral prednisone; PGE2, prostaglandin E2; SIT, specific immunotherapy; s.c. subcutaneous; TNF, tumor necrosis factor*.

These numbers illustrate quite well that the methodological issues, as described in both Task Force reports (1, 2), are still actual and need further clarification/research.

## Findings From Cross-Sectional Studies

As previously stated, the different cross-sectional studies published in the past 5 years are difficult to compare (Table 1).

Cytokines were the most studied biomarkers. Earlier studies have shown increased cytokines in EBC from children with asthma and preschool wheeze compared to healthy controls (6). These findings were reproduced in the past years for specific cytokines. Only one study compared EBC from asthmatic children with healthy controls, and found a significant increase in IL-6 (18). Within the group of asthmatic children, no difference in IL-6 levels was found between children treated with low-dose inhaled corticosteroids (ICS) compared to asthmatics treated with high-dose ICS (18). Unfortunately, no other markers of disease severity or degree of asthma control were reported, nor were other cytokines assessed.

Two other studies assessed cytokines in EBC of asthmatic children with exercise-induced bronchoconstriction (EIB) (19, 23). In a study assessing EBC biomarkers in asthmatic children with EIB, detectable levels of interleukin-4 (IL-4) were found in most but not all children who had a positive methacholine challenge test (23). However, in half of the collected EBC samples, cytokine levels were below the lower limit of detection (23). In another study, IL-16 and monocyte chemotactic protein-1 (MCP-1) were significantly higher in EBC from asthmatic children with EIB compared to asthmatics without EIB (19). However, other measured cytokines were not related to changes in forced expiratory volume in 1 s (FEV_1_) after exercise (19). A major limitation of both these studies, is the lack of EBC collection before exercise. It is therefore uncertain whether the measured differences in cytokines between subjects truly reflect an inflammatory response induced by exercise.

In a very small study in children with severe asthma (*n* = 8), IL-2, MCP-1, and two markers of airway remodeling (platelet-derived growth factor BB and tissue inhibitor of metalloproteinase 2) showed a good correlation with fractional exhaled nitric oxide (FeNO) (12). Again, there was no correlation with other cytokines, and the inter-subject variability of cytokines was great. Moreover, the variability in FeNO was also great (8.9–251 ppb), questioning the usefulness of correlating cytokines with a single or baseline FeNO measurement.

Interestingly, all the above described studies found neither detectable nor increased levels of IL-5 or IL-13, two major T helper 2 (Th2) cytokines that are considered to play a major role in allergic asthma (39). Only one recent study showed significantly higher IL-5 in atopic compared to non-atopic asthmatics (27). Furthermore, the atopic asthmatics had a significant higher blood eosinophil count, which is in line with the previous finding.

Acidity is another frequently investigated biomarker. Earlier studies have shown a decreased pH in children with severe compared to mild asthma (6). With respect to asthma control, pH was significantly decreased in a group of children with uncontrolled asthma as defined by GINA guidelines (21). However, in other recent studies, no significant difference in EBC pH was found between asthmatic and non-asthmatic children, nor between atopic and non-atopic asthma (20, 28). Nonetheless, sample sizes from both studies were small. Another potential reason could be the use of regular ICS in the majority of children in the latter study, as earlier studies have shown an increase in EBC acidity after treatment with ICS (2, 6). Conflicting results regarding acidity in EBC, for example in asthma vs. healthy, have been described in both pediatric and adult studies (7, 40). A possible explanation could be the influence of environmental factors, of which carbon dioxide seems the most important one (2, 41).

Regarding eicosanoids in EBC, earlier literature has shown mixed results. 8-isoprostane (8-IP) is increased in childhood asthma, and then particularly in children with severe asthma or with an asthma exacerbation (6). This finding was replicated in a small study in which 8-IP levels were higher in moderate persistent asthma compared to mild persistent asthma (16). Moreover, 8-IP levels in this study were higher in children having >4 exacerbations per year compared to children with 1–4 exacerbations per year (16).

In the same study, cysteinyl leukotrienes (Cys-LTs) in EBC showed a tendency to increased levels in the moderate persistent asthma group, but not a significant difference (16). Both the lower detection rate (73%) and the low sample size could be potential causes for this lack of significance. In another study performed in children living in a city with high levels of air pollution (PM_10_), Cys-LTs were higher in asthmatics compared to healthy children (17). Furthermore, Cys-LTs were higher in children with persistent asthma than in children with mild intermittent asthma (17).

An interesting finding comes from a study assessing leukotriene B_4_ (LTB_4_) in children with asthma compared to healthy controls (26). In this study, EBC was collected by using an EcoScreen 2 condenser, which is capable of partitioning exhaled breath in a large airway fraction and a small airway or alveolar fraction. LTB_4_ was only increased in the alveolar or small airway fraction, and not in the large airway fraction of EBC (26). Moreover, children with an obstructive lung function (FEV_1_ < −1.65 *z*-score) had significantly higher LTB_4_ than children without obstructive lung function (26). Unfortunately, no other eicosanoids were measured. Nevertheless, these findings are intriguing and raise the question how concentrations of other EBC biomarkers differ between the proximal and distal airways. To our knowledge, no other studies assessed fractioned EBC sampling nor its impact on various biomarkers in pediatric asthma.

With respect to asthmatic children with EIB, one study showed detectable levels of eicosanoids in children with a positive exercise challenge test, whereas this was not the case in children with a positive methacholine challenge test (23). Again, sample size was low. Two other studies assessed lipoxins in EBC. Lipoxin A4, an anti-inflammatory mediator, was increased in EIB positive asthmatic children when measured directly after exercise (24). Furthermore, it was inversely related to the degree of reduction in post-exercise FEV_1_% (24). However, in another study by the same authors investigating another anti-inflammatory mediator, Annexin A5, no differences were found directly after exercise (25). This demonstrates the contrasting findings in and between the various EBC studies.

The previously mentioned studies assessed biomarkers already known in the field, using well-known analytical methods, whereas only a few studies used other analytical methods (13, 22). In a study by Carraro et al., a metabolomic approach based on mass spectrometry was used on EBC (13). Using Bidirectional-Orthogenal Projections to Latent Structures-Discriminant Analysis, a robust model was found that discriminated very well between severe asthma and healthy controls, as well as between severe and less severe asthma (13). Sinha et al. used nuclear magnetic resonance spectroscopy on EBC (22). This method was able to differentiate children with asthma from healthy controls with a sensitivity of 80% and specificity of 75%, and identified 3 metabolomic clusters with different clinical and chemical features (22).

Although the aforementioned studies show intriguing findings, they should still be considered as part of a new or ongoing pilot project in which a single marker or analytical method is tested on EBC. Only one recent cross-sectional study tried to create a discriminative model using different EBC markers (20). In this study, a significant predictive model for asthma was created using six different biomarkers: pH, pCO_2_, pO_2_, magnesium, calcium, and urates (20). Still, diagnostic accuracy was only modest with an area under the ROC curve (AUC) of 0.698, positive predictive value (PPV) of 84%, and negative predictive value (NPV) of 39% (20).

## Findings From Longitudinal Studies

By means of the search strategy, we identified 10 longitudinal studies of which two were randomized double-blind placebo controlled trials. Keskin et al. studied the effect of montelukast on preschool wheezing, and measured eicosanoids in EBC at first visit (32). Unfortunately, EBC was not collected after start of the trial in order to evaluate the effect of intervention on EBC markers. In the study by Stelmach et al., the effect of various treatment regimens was investigated in children with asthma and EIB symptoms (35). Eicosanoids and cytokines were determined in EBC collected before, and at 8 weeks of intervention. However, comparison between groups was not possible due to low or no detectable levels of the studied biomarkers (35). In only a few subjects, detectable levels of IL-4 and eicosanoids were found after exercise (35).

The remaining eight longitudinal studies were prospective cohort studies with a varying duration of follow-up. Two of these studies determined EBC acidity in a very specific subpopulation not relevant for this current review, such as the effect of specific immunotherapy on asthma symptoms (34), and the relationship between *Mycoplasma pneumoniae* and asthma control (38).

Three studies investigated EBC collected during and after an asthma exacerbation. As stated before, increased levels of both 8-IP and Cys-LTs have been found in children with asthma, particularly during an asthma exacerbation (6). In a study investigating the effect of a single-high-dose ICS compared to oral prednisone followed by a course of 6 days high-dose ICS or oral prednisone in children with an asthma exacerbation, Cys-LTs in EBC significantly decreased in both treatment groups after both 4 h and 6 days of treatment (31). The reduction in Cys-LTs after oral prednisone is in line with earlier findings (42). With respect to the effect of ICS on Cyst-LTs in EBC, earlier studies found no reduction (43–45). However, in the study by Keskin et al. higher doses of ICS were used (single dose of 4,000 mcg nebulized fluticasone propionate followed by 1,000 mcg daily by pressurized metered dose inhaler), which could explain the positive effect (31). In the same study, no significant differences in 8-IP levels were found after 4 h or 6 days of neither high-dose ICS nor oral prednisone treatment (31). Earlier studies have shown the same results regarding the lack of effect of ICS (6), although a decrease in 8-IP after oral prednisone has been reported in the past (42).

Another group assessed acidity in EBC during and after treatment of an asthma exacerbation (29). pH was significantly lower in children with an asthma exacerbation compared to healthy controls (29). After treatment of the exacerbation (defined according to GINA guidelines), no differences were found between asthmatic and healthy children (29). The third study assessed the cytokines tumor necrosis factor (TNF) and IL-8, and a marker of airway remodeling [matrix metalloproteinase (MMP)-9] in EBC in a very small group (*n* = 4) of children with an asthma exacerbation (30). After 4 weeks of increasing the ICS dose, the concentration of TNF in EBC significantly decreased (30). However, the concentration of MMP-9 did not change, and more importantly, IL-8 was below the detection limit in all children (30).

All previous studies had a relatively short follow-up time, and only a few studies have investigated EBC over a longer follow-up period. In a study by van Vliet et al., EBC was 2-monthly collected for 12 months, and its potential in the prediction of asthma exacerbations was assessed (36). Using a model that consisted of acidity and cytokines in EBC, the predictive power was rather poor with an AUC of only 47% (36). When adding additional variables such as FeNO plus clinical characteristics and the Asthma Control Questionnaire score, the predictive power remained poor with an AUC of 59%. In another study by van Vliet et al., cytokines in EBC were 2-monthly assessed for 12 months, and correlated with asthma control (37). No significant association was found between asthma control and cytokines in EBC (37). In the same study, volatile organic compounds (VOCs) in exhaled breath were assessed by gas chromatography-mass spectrometry at the same time of EBC collection. By using a model of 15 VOCs, children with persistently controlled asthma were very well discriminated from children with persistently uncontrolled asthma during all clinical visits (AUC 86%) (37). Adding markers in EBC to this model did not lead to a better classification (37). Finally, in the study by Klaassen et al., markers in EBC were used together with exhaled VOCs and gene expression in preschool children with wheeze that were followed until school-age when a definite diagnosis of asthma was made (33). In this study of 202 wheezing preschool children, a combined model using the asthma predictive index with gene expression and exhaled VOCs showed a very accurate prediction of asthma at age 6 (AUC 95%, PPV 90%, NPV 89%) (33). However, markers in EBC did not contribute to this model (33). Unfortunately, the last few studies showed that EBC in its current state has no added value yet in a real-life clinical setting.

## Discussion

In this review, we have summarized findings of EBC research on pediatric asthma performed in the past 5 years (2013–2018). As shown above, these recent studies showed mixed results, which was also the case in reviews regarding older literature (6, 7). Moreover, study results are hardly comparable due to large heterogeneity in study population, study methods, EBC collection and methodology, EBC biomarkers, analytical methods and limits of detection. Moreover, most studies had a very low sample size. As a result, this review cannot give an univocal conclusion on whether EBC is useful in the diagnosis or monitoring of childhood asthma. Based on current technology, methods, and clinical results, it is not ready as diagnostic tool for clinical application yet.

As shown in Tables 1, 2, recent research has mainly focused on well-known biomarkers in EBC, such as cytokines, acidity, and eicosanoids. Interestingly, NO_x_ and H_2_O_2_, markers of oxidative stress that got a lot of attention in the early days of EBC research, were not incorporated in recent research. This may reflect persistent methodological problems, as illustrated by the only study that assessed EBC NO_x_, which reported a detection rate of only 48% (9). The ***rate of detection*** is also an important issue in the assessment of eicosanoids and cytokines in EBC, as values near and even below the lower limit of detection were frequently reported. However, detection rates were not reported in more than half of the studies that assessed these biomarkers (9/17, Tables 1, 2), which is a shortcoming. Because of the dilution of EBC and low concentration of EBC biomarkers, it is particularly important to have very sensitive, reliable, and reproducible assays for measurements of biomarkers. As far as we know, no special assays for use in EBC have been developed. As a consequence, different assays have been used in the various studies, whereas information regarding inter-assay variability is lacking. The measurement of pH is another good example of ***methodological heterogeneity***, as 3 different methods were used in 9 studies, of which deaeration by argon was mostly performed (5/9). The optimal method for measuring pH in EBC has been part of debate for more than a decade (41, 46), and the best methodology has yet to be determined. Hence, the wide diversity of procedures used to collect, preserve and analyze EBC persists today and continues to impede further development and standardization. Until now there is simply no standardized procedure before, during, and after condensation, not even for only one EBC biomarker (47). Moreover, the dilution of EBC with water vapor is highly variable within and between all subjects, which in turn even compromises how to express the level of a biomarker assessed in EBC appropriately (2). An interesting relatively new method to overcome the highly variable intra- and inter-individual dilution of EBC, is the so-called particles in exhaled air (PExA) technique (48, 49). This technique samples exhaled endogenous particles originating from the epithelial lining fluid in small airways, which may allow chemical quantification with better repeatability (48, 49). A first study using the PExA technique in adult asthma showed a high feasibility, even in severe asthmatics, and the potential to assess small airway dysfunction (50). Although promising, this technique is still in its early phase, and studies in children are lacking.

Another interesting finding from our review is the shift to study specific subgroups in childhood asthma, including preschool wheezing (32, 33), children with asthma and EIB symptoms (19, 24, 25, 35), mild vs. severe asthma (12–14, 16), controlled vs. uncontrolled asthma (16, 21, 37), and asthma exacerbations (29–31, 36). Most studies focused on the diagnostic potential to differentiate between two well-defined groups at a certain time-point (cross-sectional study design). Only a few studies from only one research group have used EBC longitudinally in a real-life outpatient setting, and assessed its potential in the prediction of asthma exacerbations (36), asthma control (37), or the prediction of asthma in a group of preschool wheezers (33). However, in these longitudinal studies EBC had no added value. Moreover, two of these studies simultaneously assessed VOCs in exhaled breath. Thus far, it appears that VOCs in exhaled breath has better diagnostic value than markers in EBC (33, 36). Does this mean that EBC has been outrun by other breath tests? Considering the low number of studies measuring VOCs (assessed by either mass-spectrometry or electronic nose) in pediatric asthma in the past 10 years, the answer seems no (51). However, studies using VOCs in pediatric asthma showed very promising results with very accurate discrimination from healthy controls (52, 53). Moreover, the introduction of electronic noses (eNose) may be an important step in making VOCs a useful tool for daily practice. Nevertheless, diagnostic accuracy of eNoses varies, whereas the mass-spectrometric technique seems more accurate and appropriate (54). On the other hand, the collection and analysis of exhaled VOCs suffers from the same lack of standardization and methodological issues as EBC (2).

In conclusion, despite the fact of being a non-invasive tool with high potential in (young) children, and despite the publication of many recommendations by both the ERS and ATS, the development of EBC into maturity seems to have stagnated. The main concern remains the various methodological issues before, during, and after breath condensation, as clearly shown by all methodological recommendations from the latest ERS Task Force (2). Naturally, these recommendations were published very recently and have not yet seeped through to the whole of the EBC research community. Still, a high variability is found regarding breath condenser, collection time, collected volume, prevention of salivary or environmental contamination, cleaning procedures, ambient air conditions, storage conditions, time between collection and analysis, and analytical methods. Moreover, most of these issues are not reported in recent studies. One must therefore almost conclude that in childhood asthma, EBC has not yet outgrown the status of a large pilot project, and is still hoping for a major breakthrough.

## Future Directions

We urge to recognize that the current use of EBC resembles a popular hit-and-run game, almost without clear rules or a single solution within reach. In our opinion, the necessary procedural rules or methodological standardization can only be achieved through a joint multicenter research initiative and a study design that will clearly address each step of EBC collection, preservation, processing, and analysis for each type of biomarker. Meanwhile peer-reviewed recommendations for publication of new data on EBC should be based on a thorough and obligatory presentation and description of each procedural step as mentioned.

## Author Contributions

MB and PR included the studies, extracted the data, and drafted the manuscript. QJ and ED helped to draft the manuscript. All authors read and approved the final manuscript.

### Conflict of Interest Statement

The authors declare that the research was conducted in the absence of any commercial or financial relationships that could be construed as a potential conflict of interest.
